# Association of inflammatory biomarkers with subsequent clinical course in suspected late onset sepsis in preterm neonates

**DOI:** 10.1186/s13054-020-03423-2

**Published:** 2021-01-06

**Authors:** Şerife Kurul, Sinno H. P. Simons, Christian R. B. Ramakers, Yolanda B. De Rijke, René F. Kornelisse, Irwin K. M. Reiss, H. Rob Taal

**Affiliations:** 1 Department of Pediatrics, Division Neonatology, Erasmus Medical Center, Erasmus MC, University Medical Center-Sophia Children’s Hospital, Research Neonatology (Sk-4246), PO Box 2060, 300 CB Rotterdam, The Netherlands; 2 Department of Clinical Chemistry, Erasmus Medical Center, University Medical Center, Rotterdam, The Netherlands

**Keywords:** Interleukin-6, C-reactive protein, Procalcitonin, nSOFA, Sepsis, Late onset neonatal sepsis, Neonatology

## Abstract

**Background:**

Sepsis is a major health issue in preterm infants. Biomarkers are used to diagnose and monitor patients with sepsis, but C-reactive protein (CRP) is proven not predictive at onset of late onset neonatal sepsis (LONS) diagnosis. The aim of this study was to evaluate the association of interleukin-6(IL-6), procalcitonin (PCT) and CRP with subsequent sepsis severity and mortality in preterm infants suspected of late onset neonatal sepsis.

**Methods:**

The study was conducted at the Erasmus University Medical Center–Sophia Children’s Hospital Rotterdam. Patient data from January 2018 until October 2019 were reviewed for all preterm neonates born with a gestational age below 32 weeks with signs and symptoms suggestive of systemic infection, in whom blood was taken for blood culture and for inflammatory biomarkers determinations. Plasma IL-6 and PCT were assessed next to CRP at the moment of suspicion. We assessed the association with 7-day mortality and sepsis severity (neonatal sequential organ failure assessment (nSOFA) score, need for inotropic support, invasive ventilation and thrombocytopenia).

**Results:**

A total of 480 suspected late onset neonatal sepsis episodes in 208 preterm neonates (gestational age < 32 weeks) were retrospectively analyzed, of which 143 episodes were classified as sepsis (29.8%), with 56 (11.7%) cases of culture negative, 63 (13.1%) cases of gram-positive and 24(5.0%) cases of gram-negative sepsis. A total of 24 (5.0%) sepsis episodes resulted in death within 7 days after suspicion of LONS. Both IL-6 (adjusted hazard ratio (aHR): 2.28; 95% CI 1.64–3.16; p < 0.001) and PCT (aHR: 2.91; 95% CI 1.70–5.00; p < 0.001) levels were associated with 7-day mortality; however, CRP levels were not significantly correlated with 7-day mortality (aHR: 1.16; 95% CI (0.68–2.00; p = 0.56). Log IL-6, log PCT and log CRP levels were all significantly correlated with the need for inotropic support.

**Conclusions:**

Our findings show that serum IL-6 and PCT levels at moment of suspected late onset neonatal sepsis offer valuable information about sepsis severity and mortality risk in infants born below 32 weeks of gestation. The discriminative value was superior to that of CRP. Determining these biomarkers in suspected sepsis may help identify patients with imminent severe sepsis, who may require more intensive monitoring and therapy.

## Introduction

Neonatal sepsis is a major health issue, particularly in the preterm neonatal population, where low birth weight, immature immune system and other compromising factors make it a primary cause of morbidity and death [1–5]. Late onset neonatal sepsis (LONS) occurs after three days of life and may be caused by pathogens acquired at delivery or during the course of hospital care [6]. Although in most cases LONS onset is often inconspicuous, the clinical course may be alarmingly fulminant leading to septic shock and death within hours of onset [7, 8]. Infected neonates must therefore be promptly identified, and additionally, sepsis severity should ideally be assessed at moment of onset to intensify and adapt monitoring and therapy. Several chemical biomarkers are used to diagnose and monitor disease progression in patients suspected of sepsis. Three commonly used biomarkers are: C-reactive protein (CRP), procalcitonin (PCT) and interleukin-6 (IL-6) [9]. CRP, the most frequently used laboratory test for the diagnosis of neonatal sepsis, is extensively studied in routine practice and available as point of care test [10]. However, CRP level at onset is proven not predictive of LONS diagnosis [11]. PCT is an acute phase reactant and the PCT response is more rapid than the elevation of CRP [12, 13]. IL-6 is a pro-inflammatory cytokine and increases rapidly before PCT or CRP increases in neonatal septic patients [12].

For individual patient management, it is unclear which patients are most likely to have a severe sepsis or which are least likely to survive at moment of sepsis suspicion and thus would benefit from more intensive monitoring and from alternative treatment approaches. Risk assessment would greatly aid decision-making in individual patients. Recently, a new sepsis severity score (e.g., organ dysfunction) was adapted from adults for use in preterm neonates with LONS. This score, the neonatal Sequential Organ Failure Assessment (nSOFA) score, consists of respiratory, cardiovascular and hematological criteria [14]. A high score (> 4 points out of a maximum of 15) 12 h after onset is associated with mortality [14].

Little is known about the predictive value of inflammatory biomarker responses in preterm infants suspected of LONS. Nevertheless, CRP seems the most commonly used and widely implemented biomarker. Furthermore, few studies have purposefully attempted to evaluate biomarkers that could objectively reflect the risk of mortality and sepsis severity of neonatal sepsis in neonates suspected of LONS [15, 16]. The aim of the current study is to investigate the association of inflammatory biomarkers at moment of suspicion of LONS with subsequent risk of mortality and sepsis severity in preterm infants (< 32 weeks of gestation at birth).

## Material and methods

### Study design and population

The study was conducted at the Erasmus MC University Medical Center–Sophia Children’s Hospital Rotterdam, a level IV neonatal intensive care unit (NICU). Patient data from January 2018 until October 2019 were reviewed for all neonates with signs and symptoms suggestive of systemic infection, in whom blood was taken for blood culture and for inflammatory biomarkers determinations.

### Ethical approval

The study was approved by the local ethical board of the Erasmus MC, University Medical Center. No explicit informed consent was obtained, the ethical board resumed responsibility. As part of standard care patients are asked for consent to use data for future medical research when admitted to our NICU.

### Definition of sepsis

The Department of Microbiology provided all results of blood cultures, including timing of collection and identified micro-organisms. We excluded all control blood cultures. One patient could provide multiple cases of sepsis. Sepsis diagnosis was established by the criteria defined by the NICHD Neonatal Research Network [17]. LONS was defined as a positive blood culture obtained after 72 h of life and intent to treat with antibiotics for 5 days or more. An episode of culture proven sepsis was defined as a positive blood culture due to an identified bacterial organism (including coagulase-negative staphylococci), treated with antibiotics for 5 days or more or treated for a shorter duration if death occurred during treatment [17]. Culture negative sepsis was defined as (1) C-reactive protein level greater than 10 mg/L within two days after blood culture, (2) antibiotic treatment longer than 5 days (or intention to treat longer) and (3) clinical signs of sepsis assessed by treating physician.

### Patient characteristics

The following patient characteristics were obtained from clinical charts: sex, gestational age, birthweight and postnatal age at suspicion. Data of the mother were collected from the Dutch Neonatology Register (LNR): age, hypertension, gestational diabetes, prolonged preterm rupture of membranes (pPROM), maternal fever, antenatal corticosteroid therapy and multiple birth.

### Biomarkers

The biomarkers included in the analysis were serum IL-6, CRP and PCT. Biomarker levels were measured by the department of Clinical Chemistry and were retrospectively queried from the laboratory information system. Blood samples were taken at the time of the initial sepsis suspicion (0 h).

CRP levels were measured using a turbidimetric method (C502, Cobas 8000 system, Roche Diagnostics, Rotkreuz, Switzerland). PCT and IL-6 were both measured using Electro-Chemi Luminescent Immuno Assay (ECLIA) tests (E801, Cobas 8000 system, Roche Diagnostics, Rotkreuz, Switzerland).

Plasma levels of CRP, PCT and IL-6 were routinely determined as part of a diagnostic local workup protocol whenever sepsis was suspected in infants. No other cytokines were included in this protocol. At our center, we use heart rate observation (HeRO) monitoring in preterm infants as an early warning score for LONS [18]. According to local protocol, clinicians can consider to determine chemical biomarkers when the HeRO score is increased. Blood cultures are drawn and antibiotic treatment is started when the neonate shows evident clinical signs of sepsis or when CRP, PCT or IL-6 levels are increased.

### Outcome measurements

The primary outcome was mortality within 7 days of suspicion of sepsis. The secondary outcomes were measures of sepsis severity; nSOFA score [14], need of inotropic therapy, need of mechanical ventilation and thrombocytopenia within 3 days of suspicion. The nSOFA parameters were obtained from clinical charts to calculate the nSOFA scores at time point, t = 12 h. A high nSOFA score was defined as more than 4 points out of a maximum of 15 at 12 h after onset. Inotropic support was defined as the start of inotropes after sepsis suspicion. Need for mechanical ventilation was defined as the need for intubation within 72 h after suspicion. If the patient was already intubated at moment of suspicion, this sepsis episode was excluded for analysis with this specific secondary outcome. Thrombocytopenia was defined as a platelet count < 50 × 10^9^/L.

### Statistical analysis

Statistical analysis was performed using R (R Core Team (2017), Vienna, Austria).

Categorical variables were described using absolute and relative frequencies. Continuous variables were described using medians and range (min–max) because of non-normal distributions.

A receiver operating characteristic (ROC) analysis was used to investigate the predictive value of each biomarker for 7-day mortality; a high nSOFA score at 12 h (nSOFA score > 4 [14]); inotropic support; intubation; and thrombocytopenia within 3 days. The exploratory optimal cutoff point for each biomarker was calculated using the R package ‘OptimalCutpoints.’ The Youden index was used to calculate the optimal cutoff points [19]. The Youden index calculates the cutoff point that optimizes the biomarker’s differentiating ability when equal weight is given to sensitivity and specificity. The cutoff point for predicting intubation was calculated by maximizing the specificity, to identify high risk patients to increase awareness in clinical practice. The effect of each biomarker on 7-day mortality was estimated using Cox regression models. We performed two sensitivity analyses; one additionally adjusting for previous sepsis episodes and the other for the type of infection. Due to the limited number of events, we were able to adjust for three possible confounders. All analyses were adjusted for sex, gestational age and birthweight. We investigated the 7-day survival using the Kaplan–Meier method. There is a lack of age specific reference values for these biomarkers. Based on the optimal cutoff analysis described before, the Kaplan–Meier method was used to estimate survival across groups with different levels of biomarkers at onset. The log-rank test was used to compare survival across groups with different levels of biomarkers.

The statistical analysis was performed on raw or logarithmically transformed data where appropriate. For all tests, a *p* value of less than 0.05 was considered statistically significant.

## Results

### Patient characteristics

In the study period, a total of 480 episodes of suspected LONS in 208 prematurely born patients (< 32 weeks of gestation at birth) occurred. Of these episodes, 143 (29.8%) were classified as sepsis. Gram-positive sepsis was most common (63 cases (13.1%), predominantly *Coagulase-negative Staphylococci* (CoNS) (48%) and *Staphylococcus aureus* (16%)). Gram-negative sepsis occurred less frequently (24 cases (5.0%), the most commonly found gram-negative organism was *Escherichia coli* (19%) (see Table 1)). Baseline maternal and patient characteristics are summarized in Table 1. The median gestational age of the neonates was 26 weeks and 3 days (range: 24 + 0–31 + 6), and the median birthweight was 850 (range: 445–2100) grams.

**Table 1:** Patient and mother baseline characteristics

Variable	Total episodes (n = 480)
Age of the mother (years)	30 (18–43)
Hypertension Pregnancy Disorder (PIH/PE)	107 (22.3%)
Gestational diabetes	33 (6.9%)
pPROM^a^	85 (17.7%)
Maternal Fever	22 (4.6%)
Antenatal corticosteroid therapy^b^	
None	64 (13.3%)
1 dose of betamethasone	113 (23.5%)
2 doses of betamethasone	295 (61.5%)
Singleton	386 (80.4%)
Sex (male)	249 (51.9%)
Gestational Age (weeks)	26 + 3 (24 + 0 – 31 + 6)
Post menstrual age (weeks)	29 + 1 (24 + 4 – 51 + 3)
Birthweight (grams)	850 (445–2100)
Age at Evaluation (days)	13 (4–165)
IL-6 level at onset (pg/mL)	45 (2–1396700)
PCT level at onset (ng/mL)	0.67 (0.02 – 100)
CRP level at onset (mg/L)	3.45 (0.3 – 309.0)
Mortality	24 (5%)
Type of infection	
No infection	337 (70.2%)
Culture negative	56 (11.7%)
Culture proven sepsis	87 (18.1%)
Gram positive (% of total proven sepsis)	63 (72%)
CoNS^c^	42 (48%)
*S. aureus*	14 (16%)
Others	7 (8%)
Gram negative (% of total proven sepsis)	24 (28%)
E.Coli	17 (19%)
Klebsiella	4 (5%)
Others	3 (4%)
nSOFA score	
nSOFA score at t = 12 h	0 (0–11)
Number of suspected episode	
1 suspected episode	84
2 suspected episode	46
> 2 suspected episodes	78

^a^Prolonged premature rupture of membranes

^b^Number of doses of antenatal betamethasone therapy

^c^Coagulase negative staphylococci

Values are medians (min–max range) or percentages

**Fig. 1:**
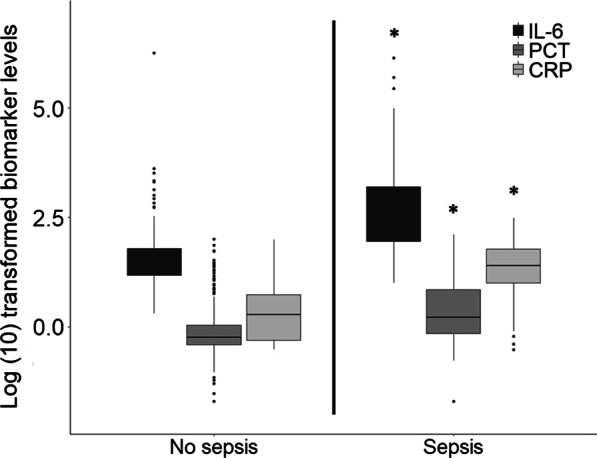
Biomarker levels at moment of suspicion. Log transformed plasma concentrations of IL-6 (pg/mL), PCT (ng/mL) and CRP (mg/L) at the time of sepsis evaluation across patients with sepsis (both culture negative and culture positive sepsis) and without sepsis. Data are presented as Log(10) transformed data because of the wide range in biomarker levels and comparability. * P < *0.001*

Figure 1 shows the plasma concentrations of IL-6, PCT and CRP at the time of sepsis suspicion (0 h). Biomarker levels in patients with sepsis differed significantly compared with patients without sepsis (p < 0.001 for all biomarkers). Excluding culture negative sepsis episodes did not affect the differences in biomarker levels when comparing patients with sepsis and without sepsis (see Additional file 1).

### Mortality

In the entire study population, a total of 24 sepsis episodes in 22 patients resulted in death within 7 days (5.0%). Log IL-6 levels were associated with 7-day mortality (adjusted hazard ratio (aHR): 2.28; 95% CI (1.64–3.16; p < 0.001), in a model corrected for birth weight, gestational age and sex. Similarly, log PCT levels were associated with 7-day mortality (aHR: 2.91; 95% CI (1.70–5.00; p < 0.001). Contrarily, log CRP levels were not associated with 7-day mortality (aHR ratio: 1.16; 95% CI (0.68–2.00; p = 0.56). Sensitivity analyses showed that these associations are independent of previous sepsis episodes and the type of infection (see Additional files 2 and 3).

**Fig. 2:**
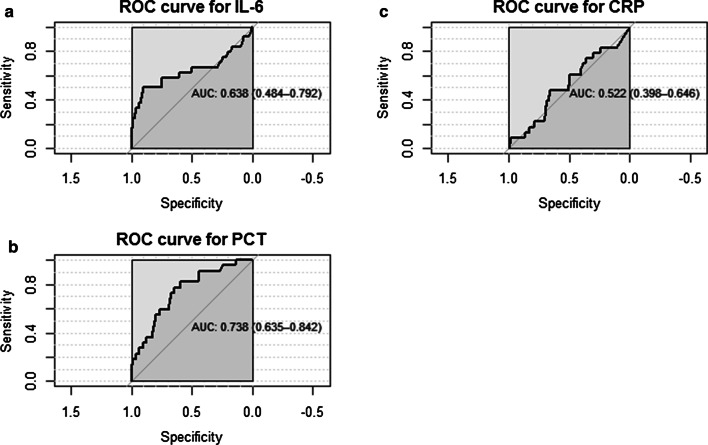
ROC curves for IL6 (**a**), PCT (**b**) and CRP (**c**) with respect to predicting 7-day mortality

**Fig. 3:**
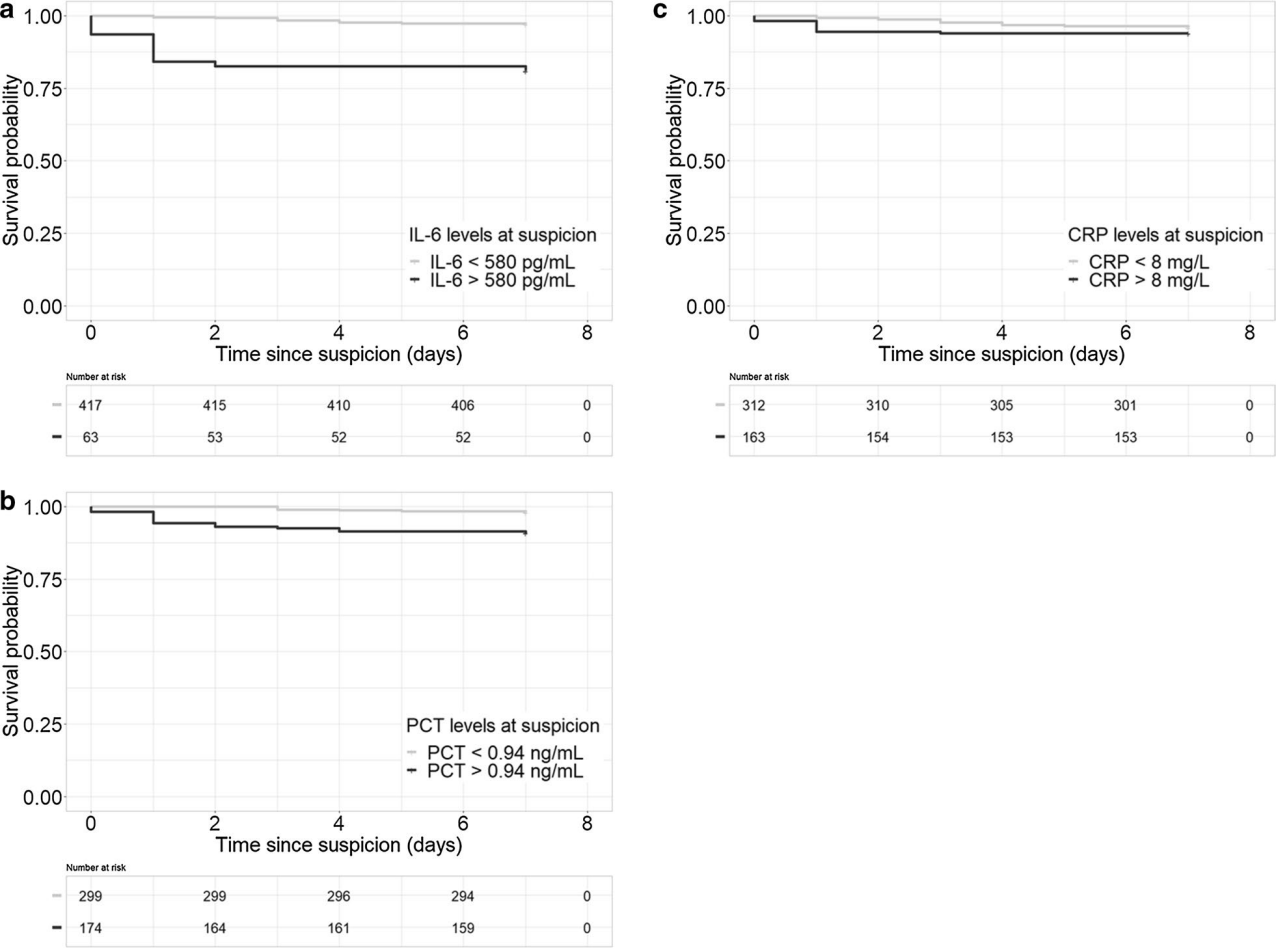
Kaplan–Meier survival curves. Primary end point of 7-day mortality according to IL-6 (**a**), PCT (**b**) and CRP (**c**) category at moment of sepsis suspicion

The area under the curve for predicting mortality was 0.64 (95% CI 0.48–0.79) for IL-6, and an exploratory cutoff point could be established at 580 pg/mL, with a sensitivity of 50.0% and a specificity of 89.9% (see Fig. 2). The area under the curve (AUC) for PCT was 0.74 (95% CI 0.64–0.84), and an exploratory PCT cutoff point could be established at 0.94 ng/mL. At this value, PCT reached a sensitivity of 77.3% and a specificity of 64.9%. The AUC for CRP was 0.52 (95% CI 0.40–0.65), and an exploratory CRP cutoff point could be established at 7.9 mg/L, with a sensitivity of 47.8% and a specificity of 65.8% (see Fig. 2). The Kaplan–Meier survival curves of the 7-day survival significantly differed across the two IL-6 categories with cutoff 580 pg/mL and the two PCT categories with cutoff 0.75 ng/mL, both p < 0.001 (see Fig. 3 panel A and B). Survival did not differ across the CRP categories with cutoff 8 mg/L at onset of sepsis, p = 0.33 (see Fig. 3 panel C).

### nSOFA score

**Fig. 4:**
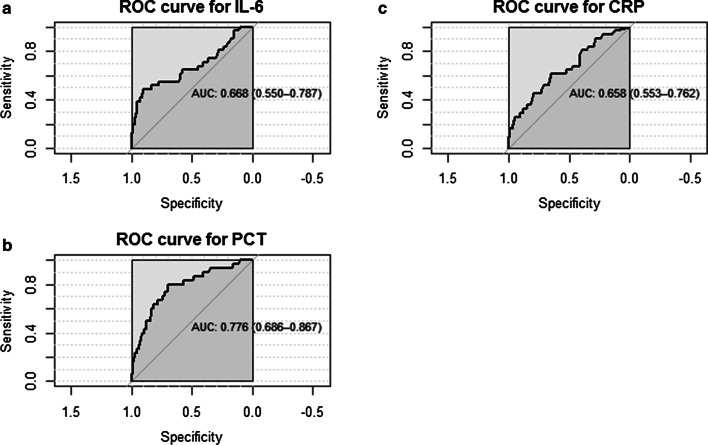
ROC curves for IL6 (**a**), PCT (**b**) and CRP (**c**) with respect to predicting a high nSOFA score (> 4) at t = 12 h after suspicion of sepsis

ROC curves and AUCs for IL-6, PCT and CRP were calculated with respect to predicting a nSOFA score above 4 at 12 h after onset. IL-6, PCT and CRP showed an AUC value of 0.668 (95% CI, 0.55–0.79), 0.776 (95% CI, 0.69–0.87) and 0.658 (95% CI, 0.55–0.76), respectively (see Fig. 4).

An exploratory IL-6 cutoff point could be established at 548 pg/mL. At this value, IL-6 reached a sensitivity of 48.3% and a specificity of 90.5%. An exploratory PCT cutoff point could be established at 1.06 ng/mL, with a sensitivity of 80.0% and a specificity of 70.2%. An exploratory CRP cutoff point could be established at 6.4 mg/L, with a sensitivity of 61.3% and a specificity of 65.3%.

### Inotropic and respiratory support and thrombocytopenia

#### Inotropic support

Log IL-6, log PCT and log CRP levels were all significantly correlated with the need for inotropic support with an aHR of 2.41 (95% CI 1.93–3.00; p < 0.001); 4.24 (95% CI 3.02–5.96; p < 0.001) and 2.0 (95% CI 1.41–2.90; p < 0.001), respectively).

ROC curves and AUC values for IL-6, PCT and CRP were calculated with respect to predicting inotropic support need, intubation and thrombocytopenia within 3 days of suspicion. AUC values with their respective 95% confidence intervals, exploratory cutoff points with sensitivity and specificity are depicted in Table 2 for each of the biomarkers. In total, 79 patients were excluded from the respiratory support analysis, due to being already ventilated at moment of sepsis suspicion.

**Table 2:** Prediction of inotropic and respiratory support and thrombocytopenia

	Total population (n = 480)	Interleukin-6	Procalcitonin	C-reactive protein
*Inotropic support*	*N = 67 cases (14%)*			
AUC		0.71	0.79	0.65
95% CI		0.63–0.80	0.63–0.80	0.56–0.73
Exploratory cutoff		118 pg/mL	1.03 ng/mL	6.3 mg/L
Sensitivity		63.1%	79.6%	58.9%
Specificity		77.8%	71.1%	65.7%
*Thrombocytopenia*	*N = 79 cases (16.5%)*			
AUC		0.77	0.83	0.79
95% CI		0.67–0.88	0.72–0.93	0.69–0.89
Exploratory cutoff		391 pg/mL	1.89 ng/mL	11.0 mg/L
Sensitivity		60.0%	79.2%	80.0%
Specificity		88.1%	82.3%	72.6%
	Total population (n = 401)			
*Respiratory support*	*N = 68 cases (17%)*			
AUC		0.67	0.72	0.59
95% CI		0.60–0.74	0.65–0.80	0.51–0.67
Exploratory cutoff		201 pg/mL	1.6 ng/mL	27 mg/L
Sensitivity		39.7%	45.3%	23.9%
Specificity		85.6%	85.8%	86.9%

Data represent AUC values with their respective 95% confidence intervals, exploratory cutoff points with sensitivity and specificity for each biomarker

## Discussion

LONS is a relevant issue in every NICU, and an early assessment of sepsis severity has the potential to improve quality of care. In this large retrospective study, we assessed the association between IL-6, PCT and CRP at onset and subsequent sepsis severity and mortality. Up until now, little has been known about the prognostic value of IL-6 and PCT for disease severity in preterm neonates suspected of LONS. Our results show that serum IL-6 and PCT, but not CRP, are associated with 7-day mortality in preterm infants suspected of LONS. IL-6, PCT and CRP at onset of sepsis were associated with various clinical measures of sepsis severity, such as need for inotropic support and mechanical ventilation, with IL-6 and PCT showing the strongest associations, with a higher discriminative value compared to CRP. To our knowledge, no other study investigated the association of these biomarkers with measures of sepsis severity. Determining these biomarkers in suspected LONS can help clinicians to identify neonates with possible imminent severe sepsis at moment of suspicion. The investigated biomarkers can be determined rapidly, usually within the matter of an hour and a half, and while the biomarker levels were related to the type of infection (e.g., gram-negative infections) they independently predicted the subsequent mortality risk. This risk assessment increases awareness and may lead to personalized monitoring. For example, peripheral arterial blood pressure measurement could be considered when high risk patients are identified, to monitor possible occurring of hypotension closely. Furthermore, they could enable personalized treatment, such as starting pentoxifylline in high risk patients [20]. Also, these biomarkers could provide a key for selecting patients at the highest risk for severe sepsis, in future clinical studies.

CRP is the most commonly available test in hospitals for neonatal sepsis [10, 11, 21, 22]. It is both used for diagnosing sepsis and monitoring disease progression. However, a recent meta-analyses by Brown and colleagues in 2255 infants showed that CRP is insensitive and nonspecific for LONS diagnosis [11]. Based on the kinetics of CRP, it is not preferred as an early prognostic and diagnostic marker; however, it has been shown that serial measurements of CRP could reliably rule-out infection 24 h after suspicion [23]. In the present study, we confirmed that CRP at onset of sepsis yields limited information on subsequent sepsis severity and risk of mortality. This is also in line with one small study by Romagnoli et al., which did not find a significant correlation of CRP levels at onset of sepsis and mortality in 39 preterm infants suspected of sepsis [24].

Little has previously been known about the prognostic value of IL-6 in neonates suspected of sepsis. We show that IL-6 can offer valuable information about the subsequent sepsis severity and risk of mortality among premature neonates suspected of LONS. Two other smaller studies showed inconsistent results of IL-6 and risk of mortality. Boskabandi et al. showed in 84 preterm and term patients that IL-6 might be valuable in predicting sepsis mortality [25], while Romagnoli et al. did not find a statistical significant difference in IL-6 levels in 39 septic preterm infants in survivors and non-survivors [24]. Studies in older pediatric patients and adults are in line with our findings; in these patients with sepsis, higher levels of IL-6 at the onset of sepsis are associated with a poorer outcome [26–28].

Serum PCT is a promising biomarker of increasing interest for detecting serious bacterial infections [29] and can be used for guiding duration of antibiotic therapy in early onset sepsis [30]. Our results suggest that PCT might also be a useful marker in predicting disease severity in LONS. However, caution is required when interpreting PCT levels [31] in an individual patient. Hahn et al. found that gestational age at birth and postnatal age had a significant effect on normal PCT levels, with more immature patients within early postnatal life having a wide range of PCT levels in a non-septic situation [31]. To the best of our knowledge, no other studies evaluated the value of PCT in predicting mortality or sepsis severity in LONS. Our study highlights a possible role of PCT in guiding clinical management.

Recently, Wynn et al. developed the nSOFA score, a sepsis severity score consisting of respiratory, cardiovascular and hematological criteria [14] and showed that a high score 12 h (> 4 out of 15) after onset of sepsis was associated with mortality. Biomarker levels of IL-6, PCT and CRP were associated with this composite outcome of sepsis severity, in which PCT had the highest diagnostic accuracy. Also, the biomarker levels were associated with each component of the nSOFA score (need for inotropic/mechanical support and thrombocytopenia). This highlights that these biomarkers at the moment of suspicion could provide information on the subsequent clinical course of the sepsis in a specific individual. To our knowledge, no other studies investigated the association of these biomarkers with measures of sepsis severity. These findings could help clinicians differentiate patients with possible severe sepsis at moment of suspicion, increase awareness for imminent clinical deterioration and also enable to personalize sepsis therapy.

There are some remarks for interpreting the study results. First, in our center we use heart rate observation (HeRO) monitoring [32] as an early warning system of sepsis. Based on changes in heart rate characteristics, we consider determining inflammatory biomarkers, possibly early in sepsis, when clinical signs are absent or non-specific. In centers without such monitoring, biomarkers could be determined later in the course of sepsis, which could affect the associations we found. For instance, after a rapid increase of IL-6 the levels also decrease rapidly and provide less information on future clinical course compared to determining IL-6 early in sepsis. This may limit generalizability of the results of our study. Second, if a patient had multiple suspected sepsis episodes we included all unique episodes independent of previous proven, culture negative or non-septic episodes. We hypothesized that the association between biomarker levels and subsequent mortality risk or sepsis severity is not modified by previous sepsis periods which a patient survived. Sensitivity analyses adjusting for previous sepsis episodes did not change the results materially. Furthermore, in daily clinical practice we would also not interpret a biomarker taking into account previous episodes. Our study also has limitations. We performed a retrospective cohort study including all patients with a gestational age < 32 weeks admitted to our hospital. We obtained many variables and outcome data from clinical charts, which may lead to misclassification due to mistakes in data entry. If misclassification would occur, we would expect this random misclassification decreasing precision of the effect estimates. We tried to limit missing data and misclassification by using automated registries for biomarker levels and blood cultures from the departments of clinical chemistry and microbiology respectively, and double data entry from the clinical charts to reduce data entry errors. Furthermore, despite being the largest cohort study investigating biomarkers levels in preterm neonates with suspected LONS, the number of events in our study is relatively small, limiting the ability to test model performance for calculated cutoff values in a validation sample.

Future research should focus on validating our findings in independent and larger populations. Furthermore, in this study we looked at short-term outcome measures; future research could focus on the relationship of these biomarkers with long-term (neurodevelopmental) outcomes measures.

## Conclusion

In conclusion, our findings demonstrate that IL-6 and PCT offer valuable information about disease severity in subsequent clinical course and mortality risk in suspected preterm LONS. Both IL-6 and PCT are more informative than CRP and could guide personalized intensive monitoring and increase awareness for imminent clinical deterioration and timely intervention.

## Supplementary information

**Additional file 1**. ** Figure**: Biomarker levels at moment of suspicion. Description of data: Log(10) transformed plasma concentrations of IL-6 (pg/mL), PCT (ng/mL) and CRP (mg/L) at the time of sepsis evaluation across patients with figure A) sepsis (only culture positive sepsis, excluding patients with culture negative sepsis) and without sepsis; figure B) sepsis (both culture negative and culture positive sepsis) and without sepsis. Data are Log(10) transformed for visualization purposes.

**Additional file 2**. **Table**: Estimates of hazard ratios of each biomarker for 7-day mortality with and without adjusting for previous sepsis episodes. Description of data: Effect estimates reflect the hazard ratio with their 95% confidence intervals. **p<0.001. Biomarker levels are Log(10) transformed plasma concentrations of IL-6 (pg/mL), PCT (ng/mL) and CRP (mg/L).

**Additional file 3**. **Table**: Estimates of hazard ratios of each biomarker for 7-day mortality with and without adjusting for type of infection. Description of data: 1Type of infection defined in 4 categories: no – culture negative – gram-positive – gram-negative sepsis. Effect estimates reflect the hazard ratio with their 95% confidence intervals. *p< 0.05 ** p<0.001. Biomarker levels are Log(10) transformed plasma concentrations of IL-6 (pg/mL), PCT (ng/mL) and CRP (mg/L).

## Data Availability

The datasets generated during the current study are not publicly available due to privacy legislation but are available from the corresponding author on reasonable request.

## Abbreviations

AUC: Area under the curve
CRP: C-reactive protein
IL-6: Interleukin-6
LONS: Late onset neonatal sepsis
nSOFA: Neonatal sequential organ failure assessment
PCT: Procalcitonin
ROC: Receiver operating characteristic
