# Supplementary material for: Association of inflammatory biomarkers with subsequent clinical course in suspected late onset sepsis in preterm neonates
Source: Crit Care. 2021 Jan 6;25:12. doi: 10.1186/s13054-020-03423-2 (PMC7788923; doi:10.1186/s13054-020-03423-2)
Supplement: Supplementary file 1 — Additional file 1. Figure: Biomarker levels at moment of suspicion. Description of data: Log(10) transformed plasma concentrations of IL-6 (pg/mL), PCT (ng/mL) and CRP (mg/L) at the time of sepsis evaluation across patients with figure A) sepsis (only culture positive sepsis, excluding patients with culture negative sepsis) and without sepsis; figure B) sepsis (both culture negative and culture positive sepsis) and without sepsis. Data are Log(10) transformed for visualization purposes. [file 13054_2020_3423_MOESM1_ESM.pdf]

Additional file 1

Figure: Biomarker levels at moment of suspicion.

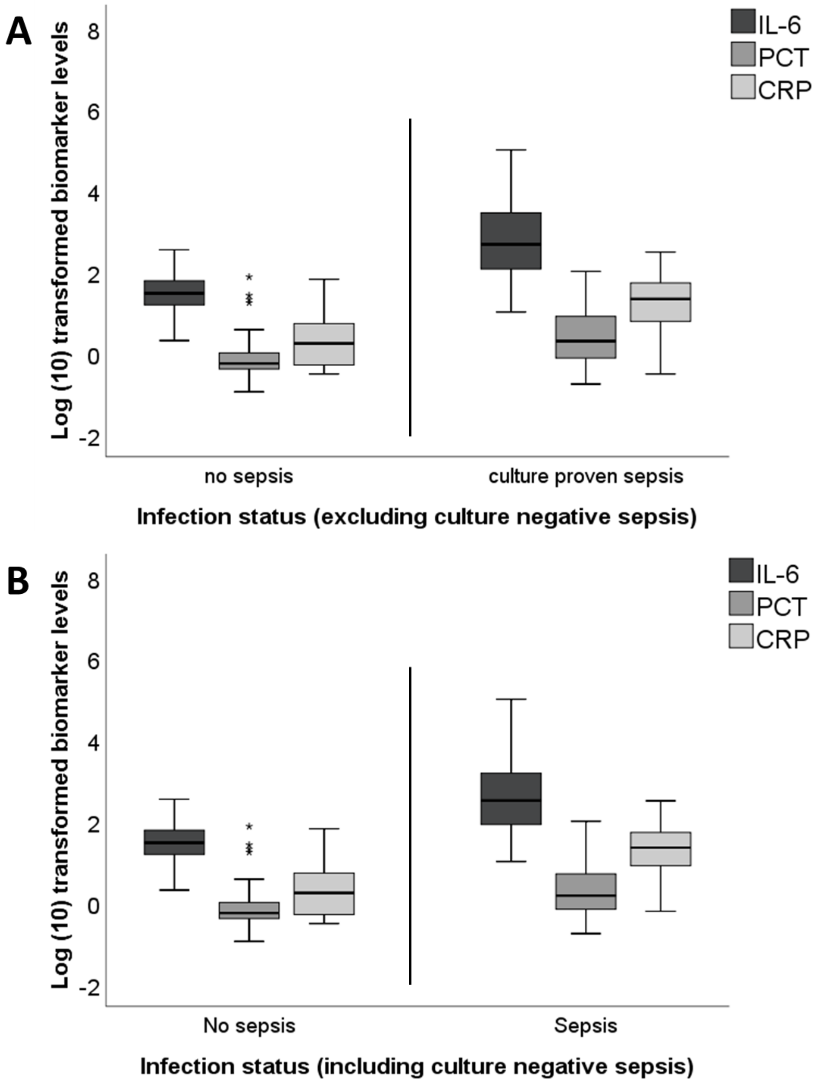

Log(10) transformed plasma concentrations of IL-6 (pg/mL), PCT (ng/mL) and CRP (mg/L) at the time of sepsis evaluation across patients with figure A) sepsis (only culture positive sepsis, excluding patients with culture negative sepsis) and without sepsis; figure B) sepsis (both culture negative and culture positive sepsis) and without sepsis. Data are Log(10) transformed for visualisation purposes.
