# Supplementary material for: Association of inflammatory biomarkers with subsequent clinical course in suspected late onset sepsis in preterm neonates
Source: Crit Care. 2021 Jan 6;25:12. doi: 10.1186/s13054-020-03423-2 (PMC7788923; doi:10.1186/s13054-020-03423-2)
Supplement: Supplementary file 2 — Additional file 2. Table: Estimates of hazard ratios of each biomarker for 7-day mortality with and without adjusting for previous sepsis episodes. Description of data: Effect estimates reflect the hazard ratio with their 95% confidence intervals. **p<0.001. Biomarker levels are Log(10) transformed plasma concentrations of IL-6 (pg/mL), PCT (ng/mL) and CRP (mg/L). [file 13054_2020_3423_MOESM2_ESM.pdf]

Additional file 2

Table: Estimates of hazard ratios of each biomarker for 7-day mortality with and without adjusting for previous sepsis episodes.

	Hazard ratio 7-day mortality	*Hazard ratio 7-day mortality additionally adjusted for previous sepsis episode*
Interleukin-6	HR 2.28 (1.64–3.16) **	HR 2.25 (1.62–3.12) **
Procalcitonin	HR 2.91 (1.70–5.00) **	HR 2.91 (1.68–5.04) **
C-reactive protein	HR 1.16 (0.68–2.00)	HR 1.34 (0.76–2.36)

*Effect estimates reflect the hazard ratio with their 95% confidence intervals. **p<0.001. Biomarker levels are Log(10) transformed plasma concentrations of IL-6 (pg/mL), PCT (ng/mL) and CRP (mg/L).*
