# Supplementary material for: Association of inflammatory biomarkers with subsequent clinical course in suspected late onset sepsis in preterm neonates
Source: Crit Care. 2021 Jan 6;25:12. doi: 10.1186/s13054-020-03423-2 (PMC7788923; doi:10.1186/s13054-020-03423-2)
Supplement: Supplementary file 3 — Additional file 3. Table: Estimates of hazard ratios of each biomarker for 7-day mortality with and without adjusting for type of infection. Description of data: 1Type of infection defined in 4 categories: no – culture negative – gram-positive – gram-negative sepsis. Effect estimates reflect the hazard ratio with their 95% confidence intervals. *p< 0.05 ** p<0.001. Biomarker levels are Log(10) transformed plasma concentrations of IL-6 (pg/mL), PCT (ng/mL) and CRP (mg/L). [file 13054_2020_3423_MOESM3_ESM.pdf]

Additional file 3

Table: Estimates of hazard ratios of each biomarker for 7-day mortality with and without adjusting for type of infection.

	Hazard ratio 7-day mortality	*Hazard ratio 7-day mortality additionally adjusted for type of infection*[1]
Interleukin-6	2.28 (1.64–3.16) **	1.76 (1.14–2.73) *
Procalcitonin	2.91 (1.70–5.00) **	2.07 (1.11–3.86) *
C-reactive protein	1.16 (0.68–2.00)	0.67 (0.38–1.20)

*[1]Type of infection defined in 4 categories: no – culture negative – Gram positive – Gram negative sepsis. Effect estimates reflect the hazard ratio with their 95% confidence intervals. *p< 0.05 ** p<0.001. Biomarker levels are Log(10) transformed plasma concentrations of IL-6 (pg/mL), PCT (ng/mL) and CRP (mg/L).*
